# Nasal adenocarcinoma induced by jaagsiekte sheep retrovirus in a juvenile goat: first report on pathological and molecular findings

**DOI:** 10.1007/s11259-026-11272-7

**Published:** 2026-05-13

**Authors:** Yesari Eroksuz, Mehmet Ozkan Timurkan, Engin Berber, Burak Karabulut, Emel Kara, Hayrunnisa Bostan-Yoru, Hatice Eroksuz

**Affiliations:** 1 Department of Pathology, Faculty of Veterinary Medicine, Firat University, Türkiye 23200 Elazig, Turkey; 2 Department of Virology, Faculty of Veterinary Medicine, Atatürk University, Erzurum, 25240 Türkiye Turkey; 3 Department of Infection Biology, Lerner Research Institute, Cleveland Clinic, Cleveland, OH 44195 USA

**Keywords:** Jaagsiekte sheep retrovirus, Enzootic nasal adenocarcinoma, Goat, PCR, Phylogenetic analysis, Retroviral oncogenesis

## Abstract

This study documents the first confirmed case of JSRV-associated nasal adenocarcinoma in a goat through a comprehensive analysis combining histopathological, immunohistochemical, and molecular techniques.

An autopsy was conducted on a 12-month-old goat exhibiting progressive nasal obstruction. Tissue samples from the nasal mass were examined histologically using H&E staining. Immunohistochemical (IHC) analysis was performed to detect JSRV capsid protein expression and to assess cellular proliferation markers (Ki-67 and PCNA). Molecular screening via PCR was carried out using primers specific for JSRV, ENTV-1, ENTV-2. The JSRV-positive PCR amplicon was sequenced and subjected to phylogenetic analysis.

PCR analysis confirmed the presence of JSRV proviral DNA (385 bp) in the tumor tissue, while results for ENTV-1 and ENTV-2 were negative. Histopathological examination identified a mixed glandular adenocarcinoma arising from the surface and glandular epithelium, with evidence of turbinate bone invasion. IHC revealed multifocal positivity for JSRV capsid antigen and indicated a high proliferation index (30–35%). Phylogenetic analysis classified the detected viral strain within the exogenous JSRV group. The infected goat originated from a herd with no documented contact with sheep.

This case provides the first conclusive evidence of JSRV-induced nasal adenocarcinoma in a goat, indicating a potential expansion of the virus’s host and tissue tropism. These findings highlight the necessity for further investigation into the viral envelope-receptor interactions and promoter elements that govern JSRV tropism.

## Introduction

Approximately 20% of animal tumors are linked to viral etiologies, involving diverse oncogenic mechanisms among viral families such as Retroviridae, Hepadnaviridae, Papillomaviridae, and Adenoviridae (Burrell et al. 2017; Mui et al. 2017; Parisi et al. 2023). Among these, Jaagsiekte sheep retrovirus (JSRV) and enzootic nasal tumor virus (ENTV), two closely related betaretroviruses, are well-established causes of respiratory tumors in small ruminants (Hofacre and Fan 2010; Sharp and DeMartini 2003). While JSRV induces ovine pulmonary adenocarcinoma (OPA), ENTV-1 typically affects sheep, whereas ENTV-2 has been identified in goats (De las Heras et al. 2019; Ortín et al. 2003).

Both JSRV and ENTV have been reported in Europe, Asia, and Türkiye, suggesting broad environmental adaptability (Can-Sahna et al. 2015; Toma et al. 2020). ENTV has a prevalence of 0.1–15%, mainly in goats aged 3–5 years, with no consistent association with sex or breed (De las Heras et al. 2019). 

The oncogenicity of JSRV is driven by its envelope (Env) protein, which activates the PI3K/Akt signaling pathway via a conserved tyrosine residue (Y590), leading to uncontrolled proliferation (Palmarini and Fan 2001; Maeda and Fan 2008). Although JSRV is typically associated with lung tumors, evidence of its extrapulmonary tropism is emerging. A notable report documented JSRV in a nasal adenocarcinoma of a sheep from an ENTV-free region, suggesting its ability to infect non-pulmonary tissues (Jahns and Cousens 2020).

To date, there have been no confirmed cases of JSRV-associated nasal adenocarcinoma in goats. Here, we present the first such case in a 12-month-old goat. The diagnosis was supported by histopathological and immunohistochemical findings, as well as PCR and phylogenetic analysis confirming the presence of JSRV. This case expands our understanding of JSRV’s host range and tissue tropism, and raises important questions about interspecies transmission and retroviral oncogenesis.

## Materials and methods

### Sample collection

A 12-month-old female Anatolian Hair goat exhibiting chronic nasal discharge, weight loss, and respiratory distress was submitted to the Pathology Department, Faculty of Veterinary Medicine, Fırat University. Autopsy was performed immediately after euthanasia. Tissue samples from the nasal cavity, skull, lungs, liver, small and large intestines and ocular globes were collected and fixed in 10% neutral-buffered formalin for histopathological and immunohistochemical analyses. Additional fresh tissues were stored at − 20 °C for molecular testing.

### Histopathology

Fixed tissues were processed using standard paraffin-embedding techniques. Sections  (3–5 μm) were stained with hematoxylin and eosin (H&E) for microscopic evaluation.

### Immunohistochemistry

The avidin-biotin complex (ABC) method was used for immunostaining. After antigen retrieval, sections were incubated overnight at 4 °C with rabbit polyclonal anti-JSRV capsid antibody (1:200). Positive detection was visualized using AEC as chromogen. Lung tissue from a JSRV-positive sheep served as positive control, and omission of primary antibody was used for negative controls.

To assess cell proliferation, Ki-67 and PCNA expression were evaluated on 5-µm sections. Antigen retrieval was achieved using citrate (pH 6.0) for Ki-67 and EDTA (pH 8.0) for PCNA. Sections were blocked with 3% hydrogen peroxide and incubated with rabbit anti-Ki-67 (AFO198) and anti-PCNA (AFO239) antibodies (Affinity Bioscience, 1:200).

### Nucleic acid extraction and PCR

Within the scope of the study, the two retroviral infections with established etiologic relevance to respiratory tract adenocarcinomas in small ruminants are jaagsiekte sheep retrovirus (JSRV) and enzootic nasal tumor virus (ENTV-1/ENTV-2). Therefore, tumour tissues were screened for JSRV and ENTV-1/ENTV-2. Neoplastic respiratory tract processes in sheep and goats most commonly manifest as pulmonary and nasal adenocarcinomas driven by oncogenic betaretroviruses pulmonary adenocarcinoma is associated with JSRV, whereas enzootic nasal adenocarcinoma is associated with ENTV-1 and ENTV-2. Although both virus groups use the same receptor (Hyal2) to enter the cell, the fact that JSRV forms tumors in the lungs and ENTV in the nasal cavity is explained by the promoters in the LTR (Long Terminal Repeat) region of the virus responding to transcription factors in different tissues (Yu et al. 2011a, b). Although retroviruses normally carries an RNA genome, it is targeted to find proviral DNA in the nucleic acid extraction because it is integrated into the host’s genome within the organism and exists as proviral DNA. For the extraction of proviral DNA prior to PCR, a kit was designed to extract both types of nucleic acids. DNA was extracted using WizPrep Viral DNA/RNA Mini Kit (WizBio, South Korea) targeting proviral DNA integrated into the host genome. PCR assays were performed to detect JSRV, ENTV-1 and ENTV-2. Specific primers and annealing temperatures are in Table 1. PCR conditions included an initial denaturation at 94 °C (5 min), 35 cycles of denaturation (94 °C, 1 min), annealing (as specified), extension (72 °C, 1 min), and final extension at 72 °C (5 min). Amplicons were visualized on 1.0% agarose gels stained with ethidium bromide and compared to a 100 bp DNA ladder.

**Table 1:** PCR primers and annealing conditions used in this study

Target virus	Primers (Forward–Revers)	Product size (bp)	Annealing Temp	Reference
JSRV	**JSRV-F**- CCGGAAAGAGATCGTACCGT **JSRV-R**- TAAGGAACACAAGCTCGGGG	385	58 °C	Mansour et al. 2019
ENTV-2	**ENTV2F**-AGCTGCTCATACTGTGGATC **ENTV2R**-GATCTTATCTGCTTATTTTCAG	822	53 °C	Incili et al. 2025
ENTV-1	**ENTV-1 F-**AGCAAGTTAAGTAACTTGAGATC **ENTV-1R** GCTTAGCCGTCCTAAAAGAG	938		

### Sequencing and phylogenetic analysis

PCR products from JSRV-positive samples were purified and sequenced.

The raw sequence data were compared with reference strain nucleic acid sequences from the gene bank using BioEdit version 7.0.5 software (Hall 1999). Phylogenetic trees were constructed using MEGA 11 (Tamura et al. 2021), incorporating sequences obtained from this study alongside those from GenBank. Known retroviral strains and out-group retroviruses from GenBank were included for comparison. The phylogenetic tree was generated using 1,000 bootstrap datasets based on the Maximum Likelihood method and the ClustalW algorithm. The evolutionary distance of phylogenetic tree was calculated using the Kimura 2-parameter model.

## Results

### Case history and clinical findings

**Fig. 1:**
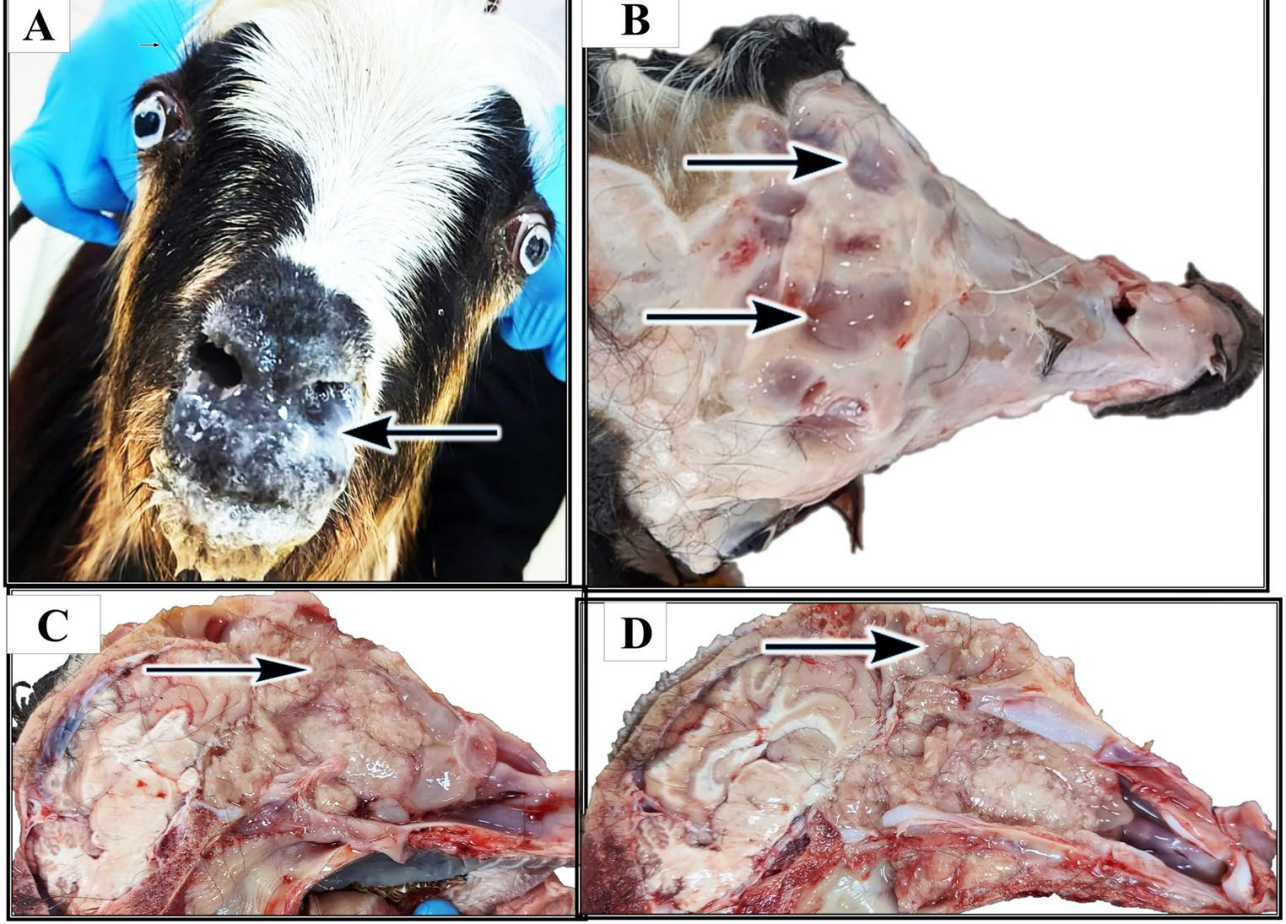
Clinical and Gross Findings of Nasal Adenocarcinoma. **A **The goat presents with bilateral nasal discharge (arrow) and noticeable swelling around the nostrils, accompanied by mild exophthalmos. **B **A dorsal view of the skull reveals multifocal bone resorption (arrow) in the frontal bones and thinning of the nasal bones. **C **Cross-section of the nasal cavity revealing multiple pale pink to white, polypoid, multilobulated tumor masses (arrow), measuring 3–5 cm in diameter. **D **The tumor masses extend into the paranasal sinuses (arrow)

A 12-month-old female Anatolian Hair goat presented with chronic bilateral nasal discharge (Fig. 1A), progressive weight loss, and dyspnea. The goat was part of a closed herd of 160 goats, with 13 animals (8.1%) exhibiting similar clinical signs including mucopurulent-to-hemorrhagic nasal exudate, open-mouth breathing, inappetence, facial edema, and reduced milk production. Among the animals showing clinical signs, five goats (3.1%) died during the 24-month follow-up period, including one juvenile and four adults. Juvenile animals exhibited a longer survival time, reaching up to 18 months, whereas adult animals showed rapid clinical deterioration, with survival limited to approximately 7–8 months. Autopsy could not be performed, and the reported information was obtained through telephone interviews with the animal owners, representing anamnesis-based findings. Importantly, the herd had no contact with sheep or other livestock. However, repeated infestations with *Oestrus ovis* larvae were reported. During a subsequent one-year period, six additional deaths occurred (3.8%), mainly among goats around two years old. According to the anamnesis data, the cause of death of the deceased animals was reported by the animal owner as tumor formations. However, since the animals were not autopsied, a definitive etiological diagnosis could not be made.

## Gross Pathological Findings

Autopsy revealed multifocal bone resorption in the frontal bones, with structural weakening of the nasal bones (Fig. 1B). Bilateral nasal obstruction was caused by polypoid, multilobulated neoplastic masses measuring 3–5 cm in diameter, characterized by pale pink to white cut surfaces (Fig.1C). These masses extended into the paranasal sinuses (Fig. 1D).

### Histopathology and immunohistochemistry

**Fig. 2:**
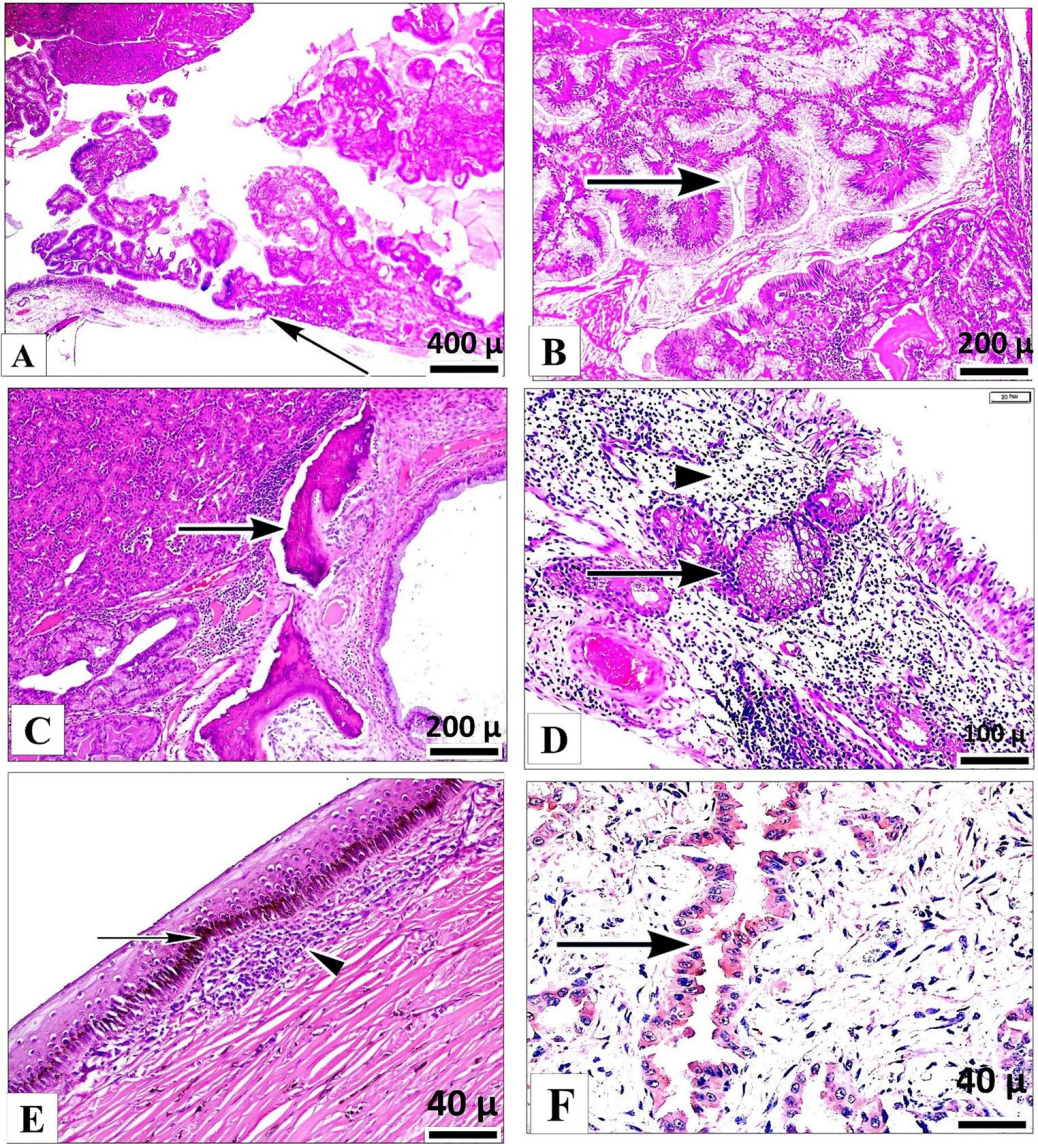
Histopathological and Immunohistochemical Characteristics of Nasal Adenocarcinoma. **A** Neoplastic cells originating from the surface epithelium (arrow), exhibiting papillary projections extending into the nasal lumen (H&E). **B** A closer view than 2A, highlighting the proliferation of a tubular (arrow) and acinar glandular structures (H&E). **C** The invasion of the tumor cells into the turbinate bone, exhibiting necrotic changes (arrow). **D** Inflammatory infiltrate (arrowhead) and glandular hyperplastic proliferations (arrow) (H&E). **E** : Mild, focal chronic keratitis characterized by melanin pigmentation in basal epithelium (arrow) and subepithelial lymphohistiocytic inflammatory infiltrate (arrow head) (H&E). **F** Immunohistochemical staining revealing positive reactivity for JSRV capsid protein in the glandular epithelium (ABC immunohistochemistry)

Histologically, the tumor originated from the surface epithelium (Fig. 2A), serous, and mucous glands. Neoplastic proliferation exhibited mixed glandular patterns, with tubular (Fig. 2B) and acinar formations. Neoplastic cells were well differentiated and exhibited low mitotic activity. The neoplastic stroma was scant and was characterized by mild interstitial edema with an accompanying inflammatory infiltrate composed predominantly of lymphocytes, plasma cells, and macrophages. In addition, glandular (arrow) and surface epithelial hyperplasia was present in the adjacent mucosa (Fig. 2C). A multifocal desmoplastic reaction was present. Chondrocytic necrosis, neovascularization, and tumor invasion were observed in the necrotic turbinate bones (Fig. 2D). Ocular tissue exhibited focal mild chronic keratitis, accompanied by epithelial pigmentation, hydropic degeneration, and lymphohistiocytic inflammation (Fig. 2E). Immunohistochemical staining for JSRV capsid protein revealed positive immunolabeling in neoplastic epithelial cells, glandular epithelium, and macrophages. Notably, immunoreactivity was more intense and consistently distributed in the glandular epithelium (Fig. 2F), while labeling in the neoplastic epithelium was less intense and limited to focal-to-multifocal areas. Immunostaining for Ki-67 and PCNA yielded consistent results, showing high proliferative activity in both mesenchymal and epithelial cells. Ki-67 expression was observed at 25%, while PCNA expression was recorded at 10%, indicating significant cellular proliferation within the tumor.

### Molecular analysis and phylogeny

**Fig. 3:**
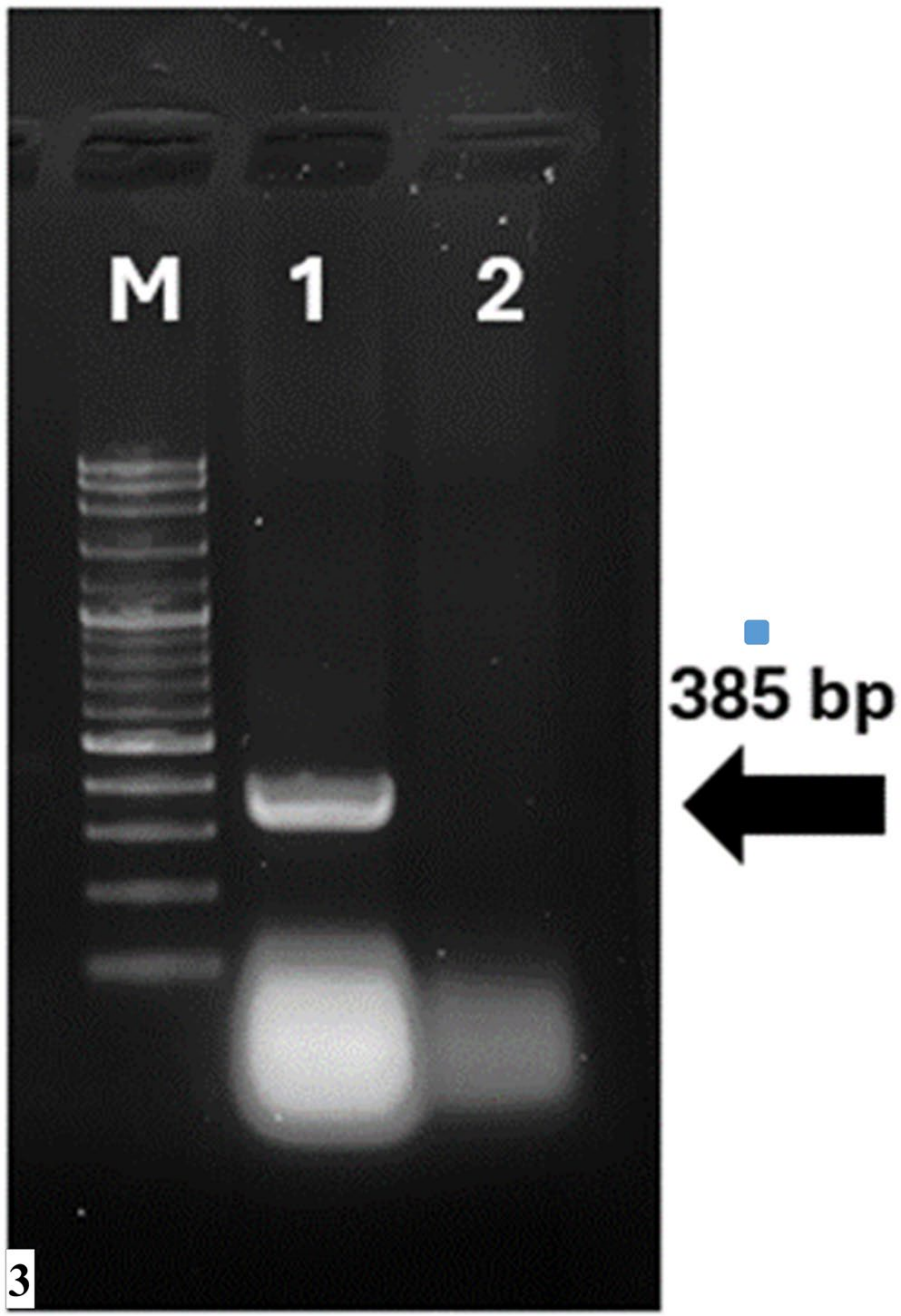
Agarose gel electrophoresis for JSRV detection tumour samples. Lane M: 100 bp ladder; Lane 1: 385 bp JSRV band; Lane 2: negative control (water)

After PCR screening for small-ruminant retroviruses included in the differential diagnosis (ENTV-1/2, and JSRV), and ENTV-1/2 were not detected, whereas JSRV was detected (Fig. 3). Agarose gel electrophoresis showed a 385-bp amplicon, which was interpreted as positive for JSRV (Fig. 3).

**Fig. 4:**
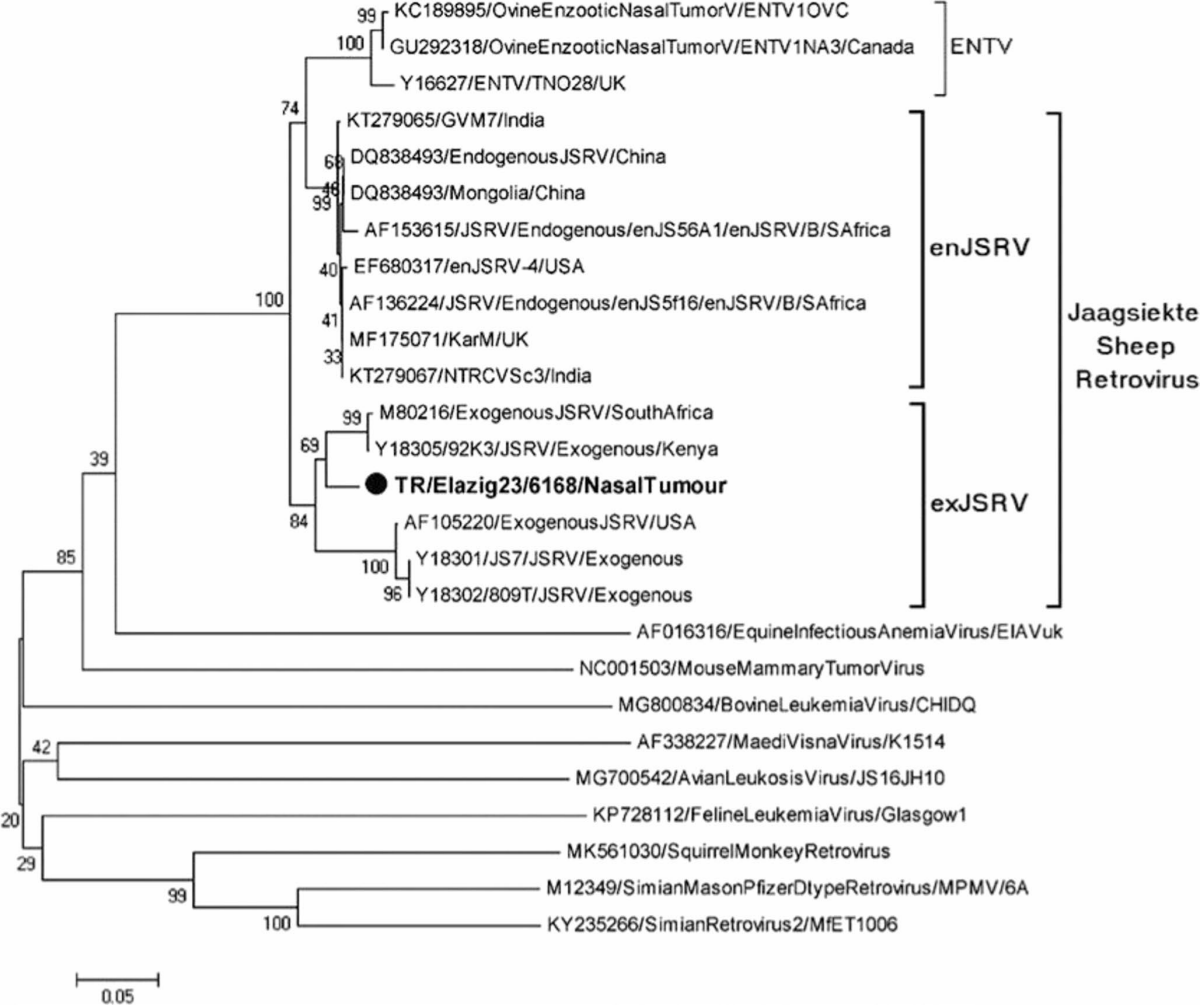
Phylogenetic tree (MEGA 11, Maximum Likelihood, K2P, 1,000 bootstraps) showing clustering of the study strain within exogenous JSRV

The PCR amplicon obtained after PCR was subjected to sequencing reaction. The raw data obtained after sequencing was organized and a phylogeny including retroviruses found in GenBank was created (Fig. 4). When Fig. 4 is examined, a phylogeny including the genera in the retrovirus family is seen. For the phylogenetic branch including ENTV and JSRV within Betaretrovirus, it is noteworthy that the strain in question is in the JSRV cluster. JSRV is found in two different forms in the organism. These are exogenic and endogenic in nature. It is noteworthy that our strain identified in the study is phylogenetically in an exogenic branch. In this case, it may indicate that the agent has a tendency to spread, that is, it exhibits an infectious character and has the potential to spread within the herd.

## Discussion

The findings presented here broaden our understanding of JSRV and ENTV, two genetically related betaretroviruses (Beachboard and Horner 2016), by providing new insights into their expanded tissue tropism, host range, and molecular mechanisms of pathogenesis. Traditionally, JSRV has been associated with ovine pulmonary adenocarcinoma (OPA), targeting alveolar type II pneumocytes and Club cells in sheep (De las Heras et al. 2019). However, recent evidence shows that JSRV can infect goats and induce pulmonary tumors, challenging its host specificity (Caporale et al. 2013). Histopathological and molecular analyses, including proviral DNA detection and comparative genomics, confirm this broader tropism and suggest that evolutionary changes or environmental factors may contribute to JSRV’s expanded pathogenicity (Alberti et al. 2002; Rosales Gerpe et al. 2019).

A key determinant of tropism is the structural difference between JSRV and ENTV. Despite sharing 95% protein sequence homology, their distinct tissue preferences—JSRV for pulmonary tissue and ENTV for nasal epithelium—are driven by differences in their long terminal repeat (LTR) promoter regions and Env protein cytoplasmic tails (McGee-Estrada and Fan 2007; Wootton et al. 2006). The LTR of JSRV contains hepatocyte nuclear factor 3β (HNF-3β) binding sites, enabling lung-specific gene expression, while the LTR of ENTV depends on CCAAT/enhancer-binding protein (C/EBP) motifs to support replication in nasal tissue (McGee-Estrada and Fan 2007). These results highlight the importance of promoter architecture in regulating tissue-specific viral replication.

Both viruses utilize HYAL2, a glycosylphosphatidylinositol-anchored receptor, for cellular entry (De las Heras et al. 2019). However, interspecies variations in HYAL2 glycosylation, tissue distribution, or nasal microenvironmental factors may limit JSRV’s nasal tropism in non-ovine hosts such as goats (Liu et al. 2003; Palmarini and Fan 2001). Murine models further underscore these limitations: while JSRV and ENTV Env proteins induce bronchoalveolar tumors in mice, nasal tumorigenesis is not observed (Wootton et al. 2006). This discrepancy likely stems from species-specific differences in immune responses, receptor interactions, and Env protein stability (Yu et al. 2011a, b). Although these models retain utility, they underscore that findings must be interpreted cautiously when extrapolated to their original host species.

JSRV’s systemic dissemination via infected lymphoid cells (Sharp and DeMartini 2003; Can-Sahna et al. 2015) and its detection in extrapulmonary sites (e.g., lymph nodes, liver) suggest a broader pathogenic reach than previously recognized. Strikingly, JSRV Env has been identified in undifferentiated lung progenitor cells (Martineau et al. 2011), suggesting a potential mechanism for latent tumor initiation. However, direct evidence of JSRV infecting nasal epithelial cells remains lacking, leaving open questions about its precise route of nasal tropism in goats.

Clinical outcomes, such as the aggressive nasal tumor progression observed in a juvenile goat in this study, may reflect age-related immune immaturity. Diminished cytotoxic T lymphocyte (CTL) activity and reduced MHC I expression in young animals could impair viral clearance, facilitating rapid neoplasia (Maginnis 2018; Mui et al. 2017). Secondary manifestations, such as chronic keratitis, may reflect local pressure of the tumor to adjacent structures, including the lacrimal ducts or nerves.

There are limited publications on JSRV in Türkiye. When these studies are examined, it is noteworthy that the virus is mostly found in the lungs and that there are limited molecular characterization studies when evaluated from a virological perspective. In the study conducted by Can Sahna et al., (2015) JSRV was shown for the first time to have an exogenous character in molecular terms and the presence of the virus was detected in extrathoracic tissues (lymph nodes, heart and liver). In another study conducted by Coskun et al. (2024), JSRV was investigated retrospectively in parafilm tissues and virus was detected in all tissues and exogenous virus was detected. In the study, while normally ENTV 1 and 2 positivity is expected in nasal tumor tissues, interestingly JSRV presence was detected. The manifestation of JSRV in nasal adenocarcinoma, where ENTV-1/2 is conventionally expected, represents a significant deviation from established viral tropism mediated by genetic plasticity and shared cellular entry mechanisms. While both JSRV and ENTV-1 share over 95% amino acid identity and utilize the Hyaluronidase 2 (Hyal2) receptor for cellular entry, they typically exhibit distinct anatomical niches—distal lungs and ethmoid turbinates, respectively—due to differences in their Long Terminal Repeat (LTR) promoter/enhancer regions. The JSRV LTR is highly responsive to lung-specific transcription factors like HNF-3 (Fox-a2), which are traditionally absent in ENTV LTRs, thereby restricting JSRV-mediated transformation primarily to type II pneumocytes and club cells. However, because the Hyal2 receptor is ubiquitously expressed throughout the respiratory tract, including the nasal mucosa, JSRV possesses the inherent capacity to infect nasal cells. The development of a nasal neoplastic process by JSRV likely arises from viral recombination or point mutations within the LTR or regulatory elements, which may relax tissue specificity and allow the oncogenic Env protein to trigger the PI3K/Akt/mTOR and Ras/MEK/MAPK pathways in the upper respiratory epithelium. This phenomenon is empirically supported by the identification of a JSRV-associated nasal adenocarcinoma in Ireland, a region where ENTV-1 has not been reported but JSRV is endemic, suggesting that in the absence of a competing betaretrovirus, JSRV may occasionally breach its typical anatomical boundaries to occupy the available oncogenic niche (Jahns and Cousens 2020). In the molecular analysis, the etiological agent was found to be exogenous JSRV. Therefore, it has been revealed that JSRV should not be forgotten in similar clinical findings, especially in small ruminant farming.

In this report, tumors were found to originate from the surface epithelium and serous/mucous glands, exhibiting mixed glandular structures with tubular and acinar formations. The neoplastic cells were well-differentiated, with minimal mitotic activity, suggesting a low-grade tumor. A comparison with the studies of Özmen et al. (2010) and De las Heras et al. (1991) reveals similar histological findings and minimal mitotic activity. Özmen et al. (2010) reported high Ki-67 and PCNA indices, indicating significant proliferation in both epithelial and mesenchymal cells. De las Heras et al. (1991) also identified tumors arising from the ethmoid region, classifying them as low-grade adenocarcinomas. These findings suggest that, despite being histologically low-grade, the proliferative activity indicates potential malignancy. Both studies emphasize that the presence of high proliferation markers, particularly Ki-67 and PCNA, could signify aggressive tumor growth. In this case, the comparatively weaker and focal-to-multifocal capsid immunoreactivity in the neoplastic epithelium, relative to the glandular epithelium, may reflect heterogeneous antigen expression related to tumor differentiation/stage and/or reduced productive viral activity within neoplastic cells.

Finally, this case highlights the **i**mportance of molecular diagnostics (e.g., PCR, sequencing) in distinguishing JSRV from ENTV, as histopathology alone lacks specificity. Future studies should prioritize in vivo models using natural hosts (e.g., goats) to validate HYAL2’s role in nasal infection and explore interspecies variations in receptor compatibility. This case expands the recognized tissue tropism and host range of JSRV, although additional cases are needed to confirm whether this represents an emerging pattern or a rare event.By bridging gaps between molecular mechanisms and clinical observations, this work advances our capacity to manage retroviral-induced neoplasia in small ruminants.

## Data Availability

All data supporting the findings are available from the corresponding author upon reasonable request.
